# Admission ferritin in STEMI predicts long-term major adverse cardiovascular outcomes

**DOI:** 10.1093/ehjopen/oeag092

**Published:** 2026-06-09

**Authors:** David Alberto Brenes-Castro, Jorge Arturo Ortega-Hernández, Ximena Latapi-Ruíz Esparza, Jorge Daniel Sierra-Lara Martínez, José Luis Briseno-De La Cruz, Héctor González-Pacheco, Rodrigo Gopar-Nieto, Alexandra Arias-Mendoza, Diego Araiza-Garaygordobil

**Affiliations:** Universidad Nacional Autónoma de México, Mexico City 04510, Mexico; Coronary Care Unit, Instituto Nacional de Cardiología Ignacio Chávez, Mexico City 14080, Mexico; Coronary Care Unit, Instituto Nacional de Cardiología Ignacio Chávez, Mexico City 14080, Mexico; Coronary Care Unit, Instituto Nacional de Cardiología Ignacio Chávez, Mexico City 14080, Mexico; Coronary Care Unit, Instituto Nacional de Cardiología Ignacio Chávez, Mexico City 14080, Mexico; Coronary Care Unit, Instituto Nacional de Cardiología Ignacio Chávez, Mexico City 14080, Mexico; Coronary Care Unit, Instituto Nacional de Cardiología Ignacio Chávez, Mexico City 14080, Mexico; Coronary Care Unit, Instituto Nacional de Cardiología Ignacio Chávez, Mexico City 14080, Mexico; Coronary Care Unit, Instituto Nacional de Cardiología Ignacio Chávez, Mexico City 14080, Mexico; Universidad Nacional Autónoma de México, Mexico City 04510, Mexico; Coronary Care Unit, Instituto Nacional de Cardiología Ignacio Chávez, Mexico City 14080, Mexico

**Keywords:** Ferritin, Inflammation, Iron deficiency, STEMI, Prognosis, MACE

## Abstract

**Aims:**

Inflammation is a key driver of atherosclerosis and acute coronary syndromes (ACS). Despite the prognostic relevance of inflammatory biomarkers in STEMI, the role of serum ferritin, an inflammation-sensitive marker of iron metabolism, remains unclear. The purpose of this study is to evaluate the association between elevated ferritin (≥300 ng/mL) and long-term major adverse cardiovascular events (MACE) in patients with STEMI.

**Methods and results:**

This is a substudy of the PHASE-MX registry, a prospective cohort of STEMI patients treated at our institution (2018–2021). Patients ≥18 years old with ferritin measurement at admission were included. The primary outcome was MACE [all-cause mortality, cardiogenic shock, recurrent myocardial infarction, and new-onset congestive heart failure (CHF)]. A total of 477 patients were included with a follow-up of 4.5 years (median of 533 days, IQR 338–1137). Median age was 59, 45.4% had hypertension, and 34.9% had diabetes. No differences in baseline characteristics were found among groups. Ferritin ≥300 ng/mL was present in 29.9% of patients and was associated with a higher risk of MACE (HR 1.43, 95% CI 1.06–1.94; *P* = 0.01), driven by all-cause mortality (HR 2.00, 95% CI 1.09–3.69) and new CHF (HR 1.46, 95% CI 1.05–2.04). This association remained significant after adjustment for inflammatory markers and GRACE score (aHR 1.48, 95% CI 1.04–2.10; *P* = 0.02).

**Conclusion:**

Elevated ferritin (≥300 ng/mL) is an independent predictor of long-term MACE in STEMI, supporting its potential role as a prognostic biomarker in ACS.

## Graphical Abstract

**Graphical Abstract:**
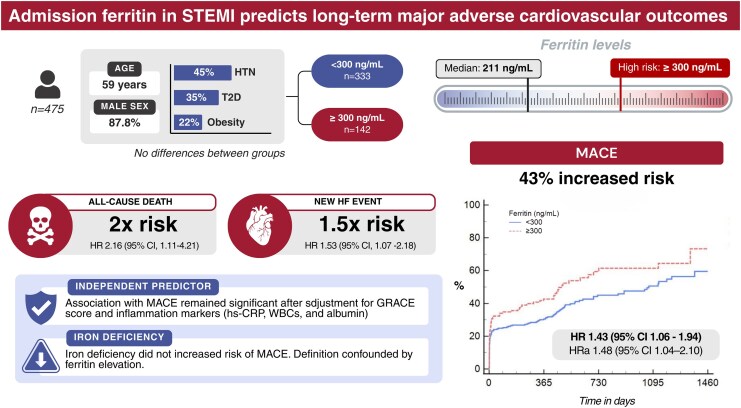

## Introduction

Inflammation is known to be involved in atherosclerosis pathophysiology, plaque destabilization, acute coronary syndromes (ACS), and adverse outcomes after an ACS.^1–3^ Even though there have been several studies evaluating the pathophysiological role of multiple inflammatory pathways and biomarkers [e.g. high-sensitivity C-reactive protein (hsCRP) and IL-6], the precise interaction between the inflammatory response and subsequent development of major adverse cardiovascular events (MACE) after an ACS is not completely understood.

Serum ferritin, though commonly used as an indicator of iron status, is also known to be elevated in response to inflammation.^4^ Furthermore, there is also preclinical evidence of ferritin's role in the iron metabolism in cardiomyocytes and potential to cause cardiomyocyte injury leading to myocardial dysfunction.^5^ Although ferritin has been associated with adverse outcomes in inflammatory conditions and ACS,^6^ evidence from large contemporary STEMI cohorts with long-term follow-up and comprehensive multivariable adjustment remains limited.

The purpose of this study is to evaluate the correlation between an initial serum ferritin level and the incidence of MACE [a composite of all-cause death, cardiogenic shock, recurrent myocardial infarction (MI) and new congestive heart failure (CHF)] in STEMI.

## Methods

This was a retrospective analysis of the PHASE-MX Registry (Evaluation of Pharmacoinvasive Strategy vs. percutaneous coronary intervention in patients with acute myocardial infarction with ST-segment Elevation at the National Institute of Cardiology in Mexico City), a prospective cohort study that included patients with STEMI presenting at Mexico City’s STEMI Network (ClinicalTrials.gov Identifier: NCT03974581). The study design details, 30 day, and long-term outcomes have been previously published.^7–9^

In summary, we included adult patients diagnosed with STEMI with at least one ferritin value determination within the first 3 days from hospital admission. Patients with a discharge diagnosis other than STEMI or those who refused to participate in follow-up were excluded. Baseline clinical characteristics, time-to-treatment, type of reperfusion therapy, in-hospital treatment, and events during follow-up were assessed by the study investigators. The study adhered to the principles of the Declaration of Helsinki, received approval from local research and ethics committees, and all participants provided informed consent for inclusion.

The primary endpoint of the study was to assess the correlation between an initial serum ferritin level ≥300 ng/mL (elevated ferritin group) and the incidence of MACE, defined as a composite of all-cause death, cardiogenic shock, recurrent MI, and new-onset CHF. Secondary endpoints included the individual components of the primary composite endpoint and bleeding events classified as Types 3–5 according to the Bleeding Academic Research Consortium criteria. Two investigators, blinded to the reperfusion strategy, independently adjudicated all outcomes. In cases of disagreement, a third blinded investigator rendered the final decision. Outcomes were defined according to international standardized definitions for clinical trial endpoints. Events during follow-up were identified through review of electronic health records and, when necessary, verified by telephone contact. Every effort was made to ascertain all clinical events; patients with missing or unverifiable outcome data were excluded from the analysis. Additional exclusion criteria included total ischaemic time >12 h (from symptom onset to treatment), unknown ischaemic time, absence of acute reperfusion, in-hospital STEMI, or discharge diagnosis other than STEMI.

### Statistical analysis

Normally distributed variables, assessed by the Shapiro–Wilk test, were expressed as mean ± standard deviation, and comparisons were made using the Student’s *t* test. Non-normally distributed variables were presented with medians and interquartile ranges (IQR), and comparisons were performed using Mann–Whitney *U* test. Categorical variables were described with frequencies and percentages and compared using the χ^2^ test or Fisher’s exact test. We described and compared quantitative variables based on their distribution by the pre-established cut-offs for ferritin (<300 and ≥300 ng/mL).

For the primary endpoint, time-to-event differences between groups were assessed using the log-rank test, and survival curves were constructed by the Kaplan–Meier method. Cox proportional hazards regression models were used—with and without adjustment for inflammatory markers [hsCRP, white blood cells (WBC), and albumin] and the GRACE score—to estimate the hazard ratio (HR) associated with ferritin ≥300 compared to <300 ng/mL (low and elevated ferritin group, respectively). In secondary analyses, individual log-rank and Cox regression models were performed for each component of the composite primary endpoint.

Ferritin, serum iron, transferrin, and total iron-binding capacity (TIBC) were obtained at hospital admission and processed in our institution’s central laboratory using validated, standardized automated assays under routine quality-control procedures; all biomarkers were measured using the same platform and procedures throughout the study period. Transferrin saturation (TSAT) was calculated with the following formula: [Serum Iron (µg/dL)/TIBC (µg/dL)*100]. Iron deficiency (ID) was defined according to standard criteria as ferritin <100 ng/mL or ferritin 100–299 ng/mL with TSAT <20%. An alternative ID definition^10^ (TSAT ≤19.8% and serum iron ≤13 µmol/L) and TSAT <20%^11^ were also explored. Because early post-STEMI events may cluster shortly after discharge, we performed exploratory short-term analyses of the primary composite endpoint at 30 and 90 days and a landmark analysis at 30 days following the index admission. Additionally, due to substantial loss of follow-up beyond 2.5 years, we conducted a *post hoc* analysis to a restricted follow-up of 2.5 years for the primary composite endpoint. Finally, the association between ferritin as a continuous variable and the primary composite outcome was modelled using restricted cubic splines with four knots placed at standard percentiles. This allowed for a flexible assessment of non-linear trends while minimizing the influence of outliers at the extremes of the distribution.

All statistical analyses were conducted using STATA version 14.1 (StataCorp, College Station, Texas), GraphPad Prism version 10.0 (GraphPad Software, LLC), MedCalc version 23.0.5 (MedCalc Software Ltd), and SAS Viya (SAS Institute Inc, 2025).

## Results

### Baseline characteristics

**Figure 1:**
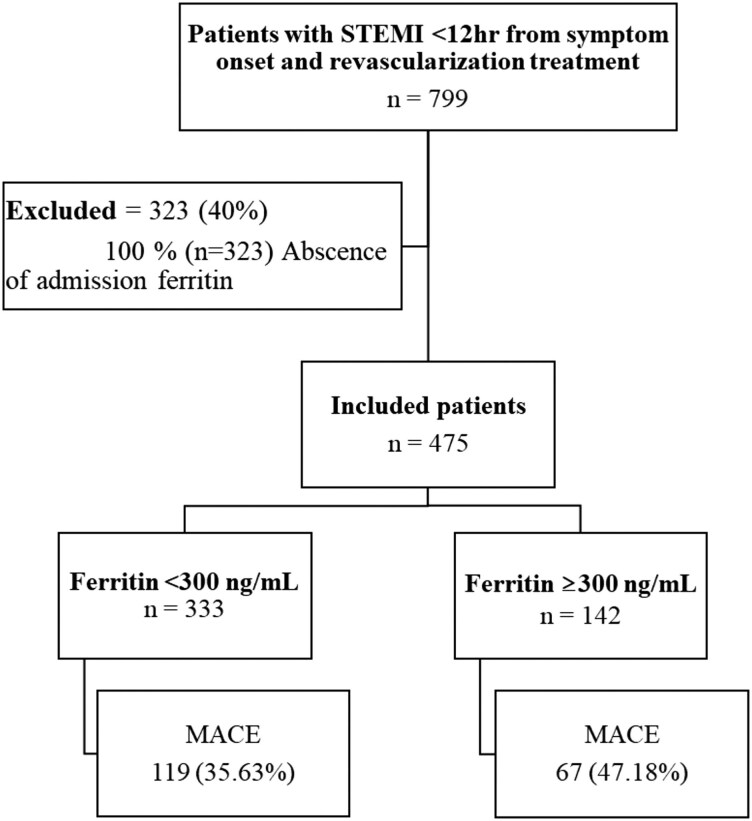
Flowchart. Patient selection process. n, number; MACE, major adverse cardiovascular events.

A total of 475 patients were included in this study. Details of patient selection can be seen in *Figure 1*. The median age was 59 years, with a male majority (87.8%). The prevalence of hypertension was 45.4%, type 2 diabetes (T2D) 34.9%, and obesity 22.3%. Specifically, the median ferritin level was 211 ng/mL, hsCRP 6 mg/L, leukocyte count 12 103/uL, and albumin 3.7 g/dL. Additional baseline characteristics are shown in *Table 1*. No statistically significant differences were found between low and elevated ferritin groups in terms of comorbidities, previous MI, clinical severity by GRACE score, or inflammatory markers hsCRP, leukocytes, and albumin. The median time from admission-to-ferritin was 1 day (IQR 0–2 days). Ferritin values were 151.9 ng/mL and 458.4 ng/mL for the low and elevated ferritin groups, respectively (*Table 1*).

**Table 1:** Baseline characteristics of the population. Ferritin groups <300 and ≥300 ng/mL

Variable	Total (*n* = 475)	Ferritin <300 ng/mL (*n* = 333)	Ferritin ≥300 ng/mL (*n* = 142)	*P*
Age, years	59 (52–65)	59 (52–65.5)	56.5 (50–64)	0.09
Male, *n* (%)	418 (87.8)	298 (89.2)	98 (89.9)	0.83
**Comorbidities and past medical history, *n* (%)**				
T2D	166 (34.9)	114 (34.2)	45 (31.9)	0.62
Hypertension	216 (45.4)	148 (44.3)	68 (48.2)	0.43
Dyslipidaemia	94 (19.7)	67 (20.1)	25 (17.7)	0.54
CKD	10 (2.1)	6 (1.8)	4 (2.8)	0.47
Obesity	106 (22.3)	70 (20.9)	36 (20.5)	0.27
Smoking	210 (44.2)	147 (44.0)	63 (44.6)	0.89
Previous MI	48 (10.1)	30 (8.9)	18 (12.7)	0.21
Previous HF	4 (0.8)	4 (1.2)	0 (0)	0.19
Atrial fibrillation	2 (0.4)	2 (0.6)	0 (0)	0.35
**Clinical presentation**				
GRACE score (IQR)	123 (99–147)	122 (98–146)	125 (101–148.5)	0.39
Haemoglobin (IQR), g/dL	15.6 (14.5–16.8)	15.6 (14.5–16.9)	15.6 (14.4–16.4)	0.58
Leucocytes (IQR), 10^3^/uL	12 (9–14)	12 (9–14)	12 (9–14)	0.70
Glucose (IQR), mg/dL	156 (124–226)	156 (124–225.3)	153 (121–235)	0.62
Creatinine (IQR), mg/dL	1.0 (0.8–1.1)	1 (0.8–1.1)	1 (0.8–1.2)	0.76
NTpro-BNP (IQR), pg/mL	786 (219–2821)	786 (241–2848)	776.5 (151.8–2791)	0.40
hsCRP (IQR), mg/L	6 (3–26)	6 (2.5–24)	7 (3–26.7)	0.40
Albumin (IQR), g/dL	3.7 (3.4–3.9)	3.7 (3.4–3.9)	3.7 (3.5–4.7)	0.25
Ferritin (IQR), ng/mL	211 (119.9–348)	151.9 (91–221)	458.4 (377.8–642.8)	<0.0001
Iron (IQR), μmol/L	57.9 (34.7–84.9)	59.8 (37.7–92.3)	47.1 (27.1–73.5)	<0.0001
TSAT (IQR), %	20.5 (13.8–29.7)	21.0 (14.0–30.0)	18.9 (12.9–27.0)	<0.0001
TIBC (IQR), mcg/dL	271.2 (236.6–305–6)	278.1 (246.4–314.0)	251.2 (208.7–278.1)	0.58

Statistical tests: χ^2^ and Mann–Whitney.

*n*, number; IQR, interquartile range; HbA1C, glycated haemoglobin; hsCRP, high-sensitivity C-reactive protein; CKD, chronic kidney disease; T2D, type 2 diabetes; TIBC, total iron-binding capacity; TSAT, transferrin saturation.

### Primary outcome

**Figure 2:**
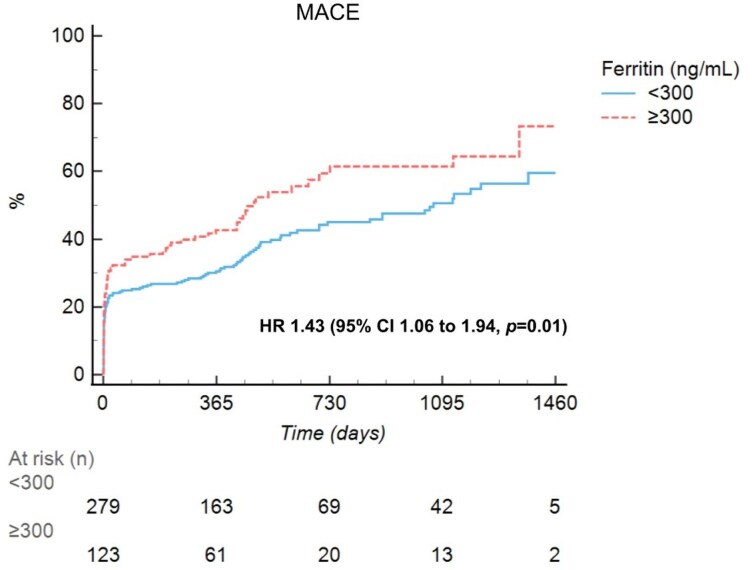
Kaplan–Meier curve of MACE at 4.5 years. Ferritin <300 and ≥300 ng/mL. MACE, major adverse cardiovascular events; n, number. Statistical analysis: Cox proportional hazard.

During a mean follow-up of 4.5 years (median of 533 days, IQR 338–1137) (see Supplementary material online, *Figure S1*), a total of 186 events of MACE occurred, 119 (35.6%) in the low-ferritin groups, and 67 (47.1%) in the elevated ferritin group (*P* = 0.018). The risk of the primary endpoint was statistically increased (HR 1.43, 95% CI 1.06–1.94, *P* = 0.01) in patients with elevated ferritin values. An adjusted analysis for albumin, NTpro-BNP, hsCRP, leukocytes, and GRACE score yielded an HR of 1.47 (95% CI 1.03–2.08) (*Table 2*, Supplementary material online, *Tables S1* and *S2*, and *Figure 2*).

**Table 2:** Number of events and risk for the primary outcome of composite MACE and individual outcomes

Variable	Total (*n* = 475)	Ferritin <300 ng/mL (*n* = 333)	Ferritin ≥300 ng/mL (*n* = 142)	*P*	HR (95% CI)	*P*
MACE	186 (39.07)	119 (35.63)	67 (47.18)	0.018	1.43 (1.06 a 1.94)	0.01
Death	42 (8.82)	23 (6.89)	19 (13.3)	0.022	1.99 (1.08 a 3.61)	0.02
New CHF	152 (31.93)	19 (20.65)	133 (34.73)	0.009	1.47 (1.06 a 2.04)	0.02
Reinfarction	27 (5.67)	20 (6.01)	7 (4.93)	0.642	0.90 (0.38 a 2.14)	0.82
Cardiogenic shock	49 (10.29)	29 (8.68)	20 (14.08)	0.076	1.60 (0.90 a 2.84)	0.10
Mayor bleeding	32 (6.72)	20 (6.01)	12 (8.45)	0.330	1.60 (0.78 a 3.30)	0.19

Statistical analysis: χ^2^ and Cox proportional hazard.

CHF, congestive heart failure; MACE, major adverse cardiovascular events.

### Secondary outcomes

#### MACE risk at 30 days, 90 days, and 2.5 years

Risk analysis indicated ferritin levels at ≥300 ng/mL associated with HRs for MACE at various time intervals: 30 days (HR 1.47, 95% CI 0.99–2.17, *P* = 0.051), 90 days (HR 1.52, 95% CI 1.05–2.22, *P* = 0.029), and 2.5 years (HR 1.53, 95% CI 1.13–2.07, *P* = 0.006) (see Supplementary material online, *Figure S2*). A landmark analysis found an HR of 1.50 (95% CI 0.95–2.37, *P* = 0.081) after the first 30 days (see Supplementary material online, *Figures S3* and *S4*).

#### Prognostic value of baseline ferritin for individual MACE outcomes

Analysis of individual MACE outcomes showed higher events in the ≥300 ng/mL group, with significant statistical association for all-cause mortality and new heart failure (HF). Primary outcome was mainly driven by an increased risk of all-cause mortality (HR 2.16, 95% CI 1.11–4.21, *P* = 0.023) and new HF (HR 1.53, 95% CI 1.07–2.18).

#### MACE risk by ferritin subgroups: <100, 100–299, and ≥300 ng/mL

**Figure 3:**
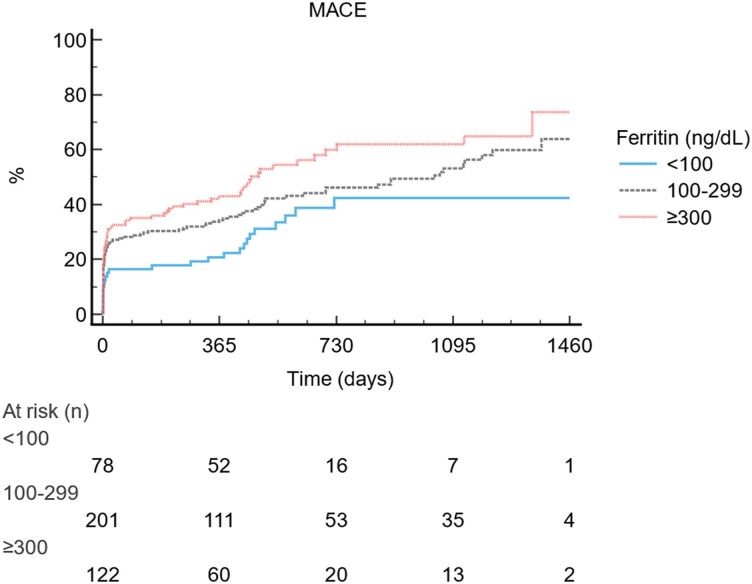
MACE at 5 years. Kaplan–Meier curve of MACE at 5 years follow-up for ferritin groups <100, 100–299, and ≥300 ng/mL. MACE, major adverse cardiovascular events; n, number. Statistical test: χ^2^ and log-rank.

Exploratory comparisons among different baseline ferritin levels revealed that dyslipidaemia was the only comorbidity with statistical differences between groups. Higher incidence of MACE, death, new CHF, and cardiogenic shock events were observed in the ≥300 ng/mL group. MACE risk was highest in the 100–299 and ≥300 ng/mL groups compared to <100 ng/mL, with HRs of 1.46 (95% CI 1.01–2.13) and 1.93 (95% CI 1.27–2.93), respectively (*P* = 0.01) (*Figure 3*, *Table 3*).

**Table 3:** Risk of the primary outcome of composite MACE at 4.5 years. Ferritin groups <100, 100–299, and ≥300 ng/mL. Statistically significant difference for groups 100–200 and ≥300 compared to <100 ng/mL

	MACE HR (95% CI)		
Ferritin (ng/mL)	<100	100–299	≥300
**<100**	—	1.46 (1.01 a 2.13)^a^	1.93 (1.27 a 2.93)^a^
**100–299**	0.68 (0.46 a 0.98)	—	1.31 (0.93 a 1.84)
**≥300**	0.51 (0.34 a 0.78)	0.75 (0.54 a 1.06)	—

MACE, major adverse cardiovascular events; n, number.

Statistical test: χ^2^ and log-rank.

^a^p = 0.01 vs. ferritin <100 ng/mL.

#### MACE risk with iron deficiency

Four hundred forty-five patients had a complete iron profile on admission and were included for this analysis. Lower levels of serum iron and TSAT were found on the group with elevated ferritin, and no difference was found for haemoglobin and TIBC between groups.

**Figure 4:**
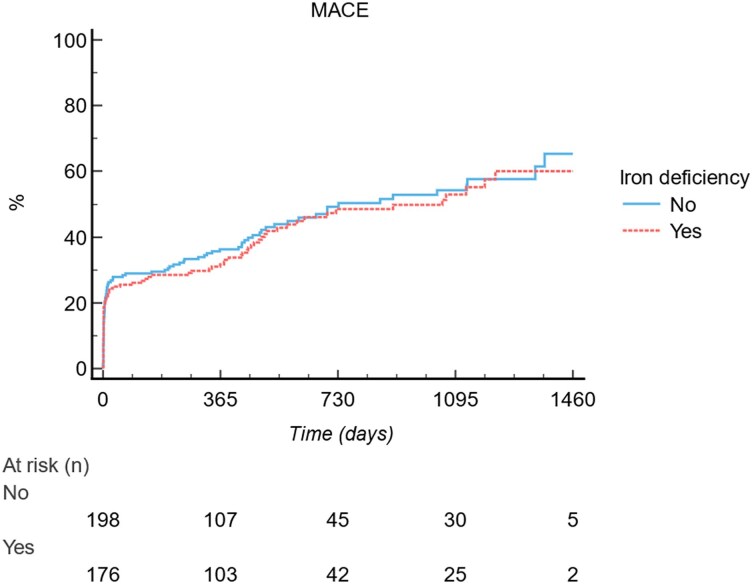
MACE composite outcome curve at 4.5 years for iron deficiency. Groups without iron deficiency and with iron deficiency. MACE, major adverse cardiovascular events; n, number. Statistical test: Cox proportional hazard.

The overall prevalence of ID according to the standard criteria was found in 45.2% of the patients; this prevalence varied significantly between the different definitions used (*Table 4*). According to the standard ID definition, no significant difference was observed for MACE risk at 1 or 4.5 years with a HR of 0.93 (95% CI 0.69–1.26, *P* = 0.67) and 0.93 (95% CI 0.69–1.26, *P* = 0.67) with or without ID, respectively (see Supplementary material online, *Figure S5* and *Figure 4*). However, TSAT <20% was associated with a HR of 1.36 (95% CI 1.01 to 1.83, *P* = 0.04) for MACE at 4.5 years.

**Table 4:** Iron deficiency prevalence

Variable	Total (*n* = 445)	Ferritin <300 ng/mL (*n* = 313)	Ferritin ≥300 ng/mL (*n* = 132)	*P*
ID definition^a^ (%)	201 (45.2)	201 (100)	0 (0)	<0.0001
ID alternate definition^b^ (%)	9 (2)	5 (55.6)	4 (44.4)	0.32
TSAT <20% (%)	219 (49.2)	149 (68)	70 (32)	0.29
Iron ≤13 μmol/L (%)	8 (1.8)	4 (50)	4 (50)	0.20
Ferritin <100 ng/mL (%)	92 (19.3)	92 (100)	0 (0)	<0.0001

n, number; TSAT, transferrin saturation. Statistical tests: χ^2^.

^a^Ferritin <100 ng/mL or ferritin between 100 and 299 ng/mL and TSAT <20%.

^b^TSAT ≤19.8% and serum iron ≤13 μmol/L was also used for analysis.

#### MACE risk across the ferritin spectrum

**Figure 5:**
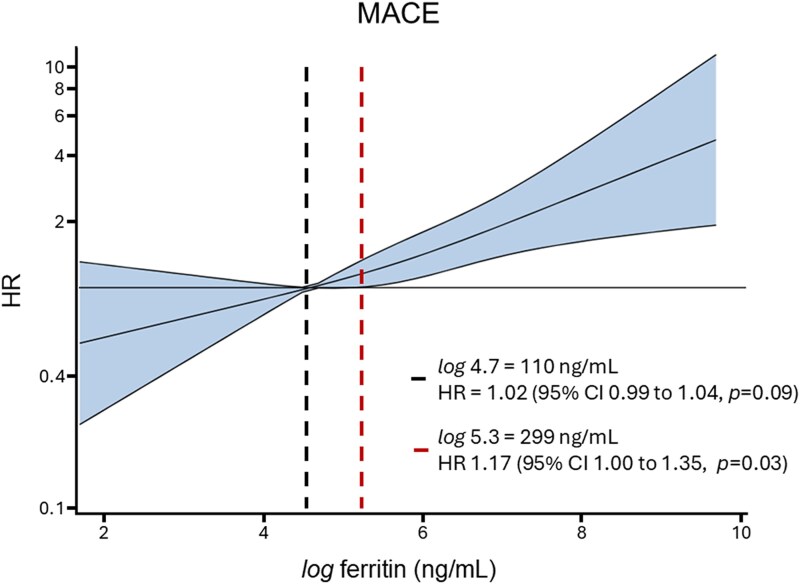
Full cohort spline analysis of MACE risk (vertical axis) for ferritin log(n) (horizontal axis). Inflection point at 110 ng/mL [4.7 log(n)] = HR of 1.02 (95% CI 0.99–1.04, *P* = 0.09). Risk onset at 299 ng/mL [5.3 log(n)] ferritin = HR of 1.17 (95% CI 1.00–1.35, *P* = 0.03). MACE, major adverse cardiovascular events; log, natural logarithm. Statistical test, Cox proportional hazard.

**Figure 6:**
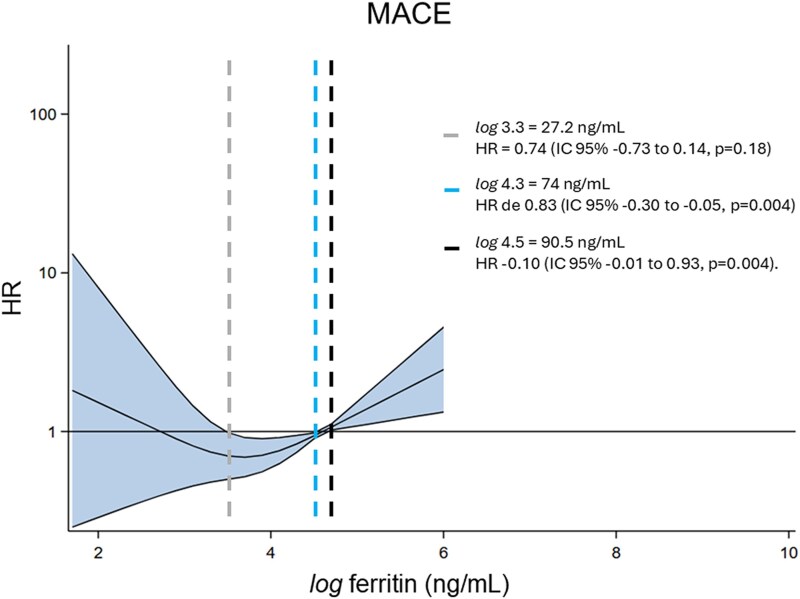
Spline curve of patients with iron deficiency for MACE risk (vertical axis) by log(n) ferritin (horizontal axis). Inflection point at 90.5 ng/mL [4.5 log(n)] = HR of −0.10 (95% CI −0.01 to 0.93, *P* = 0.004). Protection onset at 74 ng/mL [4.3 log(n)] ferritin = HR of 0.83 (95% CI −0.30 to −0.05, *P* = 0.004). Loss of protection at 27.2 ng/mL [3.3 log(n)] ferritin = HR of 0.74 (95% CI −0.73 to 0.14, *P* = 0.18). MACE, major adverse cardiovascular events; log, natural logarithm. Statistical test: Cox proportional hazard.

It was found that the inflection point between protection and risk occurred at 110 ng/mL [4.7 log(n)] of ferritin, with a HR of 1.02 (95% CI 0.99–1.04, *P* = 0.09), achieving statistical significance for hazard at 299 ng/mL [5.3 log(n)] of ferritin, with a HR of 1.17 (95% CI 1.00–1.35, *P* = 0.03) (*Figure 5*). Similarly, the same analysis was performed for patients with ID. The inflection point between protection and risk was found at 90.5 ng/mL [4.5 log(n)] with a HR of −0.10 (95% CI −0.01–0.93, *P* = 0.004); the onset of protection occurred at 74 ng/mL [4.3 log(n)] with a HR of 0.83 (95% CI −0.30 to −0.05, *P* = 0.004); and again, the loss of protection was observed at 27.2 ng/mL [3.3 log(n)] of ferritin with a HR of 0.74 (95% CI −0.73 to 0.14, *P* = 0.18) (*Figure 6*).

## Discussion

The results of this study shows that elevated ferritin levels (≥300 ng/mL) at STEMI presentation independently predict adverse cardiovascular outcomes in patients undergoing revascularization, with a 49% increased risk of MACE over 4.5 years of follow-up (median of 533 days, IQR 338–1137). These findings highlight ferritin as a potential clinically relevant prognostic biomarker in STEMI and provide new insights into the interplay of inflammation, iron metabolism, and cardiovascular outcomes in this population.

This cohort of STEMI patients represents an adequate sample with demographic and clinical characteristics reflective of contemporary STEMI populations. The prevalence of comorbidities including hypertension and T2D appropriately represents the population of interest (*Table 1*). Importantly, groups stratified by ferritin levels showed no significant differences in baseline comorbidities such as T2D or obesity nor in established markers of disease severity or inflammation including GRACE score, WBCs, or hsCRP. This balance strengthens the validity of the observed associations and minimizes potential confounding. Nonetheless, several baseline findings warrant further discussion. First, despite obesity and T2D being known to be associated with a pro-inflammatory state, these factors did not determine ferritin elevation in our cohort, suggesting that the inflammatory response to acute myocardial injury may override chronic low-grade inflammation derived by these risk factors. Second, hypoalbuminaemia—often indicative of chronic malnutrition and associated with ID anaemia—was not significantly more prevalent in the low-ferritin group, further supporting that ferritin elevation reflects acute inflammatory and pathophysiological processes rather than chronic nutritional states. Third, the median ferritin level in our cohort (211 ng/mL) was consistent with observations from similar ACS (236 ng/mL) and STEMI (186 ng/mL) populations.^11,12^ However, ferritin levels in our ‘elevated’ subgroup approached 500 ng/mL, highlighting a profound state of inflammatory activation in this specific subset of patients.

The 49% increased risk of MACE associated with ferritin ≥300 ng/mL persisted after adjustment for both inflammatory biomarkers and clinical severity variables, demonstrating independent prognostic value. The selection of covariates was guided by their established clinical relevance and findings from a landmark study evaluating inflammation-based risk scores in ACS.^13^ Temporal analysis revealed a distinctive pattern of risk across follow-up periods. Elevated ferritin at presentation was associated with adverse outcomes at 30 days (trend towards significance, *P* = 0.51), 90 days (*P* = 0.029), and 2.5 years (*P* = 0.006), while 1 year outcomes did not show significant associations. This temporal pattern suggests two key observations. First, ferritin's effect on MACE appears to be bimodal, with increased risk in the early post-MI period (30–90 days) and after 2.5 years. Second, as shown in landmark analysis (see Supplementary material online, *Figure S3*), the initial inflammatory and myocardial injury burden within 30 days contribute to sustained myocardial dysfunction and long-term cardiovascular risk. This pattern aligns with the concept of failed resolution of acute inflammation, representing a ‘legacy effect’ of the index myocardial injury and transition to a chronic inflammatory response.

Consistent with patterns observed for other inflammatory biomarkers in ACS and in line with other small observational studies evaluating ferritin,^6^ the primary composite MACE outcome was driven predominantly by all-cause mortality and incident HF, with 99 and 47% increased risk, respectively. The proposed pathophysiology linking elevated ferritin to adverse outcomes can be explained by the following mechanisms. Briefly, ferritin may function both as an end-product of inflammation (analogous to hsCRP) and as a pro-inflammatory stimulus through activation of inflammatory signalling pathways.^4,5,14,15^ Additionally, dysregulated iron metabolism within cardiomyocytes secondary to ischaemia-reperfusion injury (IRI) can enhance ferritin release,^10,15^ and when cellular iron-buffering capacity of ferritin is exceeded, ferroptosis—a form of iron-dependent regulated cell death—may ensue, further amplifying the inflammatory response and tissue injury.^16,17^ Regarding the specific association with development of HF, it likely reflects the cumulative impact of inflammatory-mediated myocardial damage, ferroptosis, and IRI, creating a substrate that leads to progressive ventricular dysfunction and ultimately HF.

Notably, ferritin was not associated with bleeding outcomes in this cohort. This finding may have clinical relevance for risk stratification in patients with elevated ferritin requiring anticoagulation or prolonged dual antiplatelet therapy following AMI. However, this specific clinical scenario was not systematically evaluated and requires validation in dedicated studies.

A dose–response relationship between ferritin levels and MACE risk was evident across ferritin categories (<100, 100–299, and ≥300 ng/mL) and confirmed by restricted cubic spline analysis (*Figure 5*), which corroborated the pre-specified 300 ng/mL cut-off of this study. However, this relationship between ferritin, ID, and prognosis in this population has important caveats. Despite nearly half of patients meeting criteria for ID, it was not associated with prognosis. These findings generate several important hypotheses. First, higher ferritin levels after AMI correlate with progressively higher MACE risk. Second, ID defined by ferritin neither confers protection nor increases risk in the acute post-MI setting. Third, the standard ID definition (i.e. ferritin-based definition) commonly employed in HF trials may be confounded by acute myocardial injury and inflammation, leading to misclassification.

Applying alternative ID definitions,^10,11^ ID prevalence in our cohort ranged from <2 to 49% (*Table 4*), highlighting major classification variability. Similar discrepancies were reported in other STEMI cohorts,^11^ where ID prevalence varied across definitions (49.0, 24.2, 57.4, and 76% vs. 45.2, 19.3, 49.2, and 1.8% using standard criteria, ferritin <100 ng/mL, TSAT <20%, and serum iron ≤13 µmol/L, respectively). In our study, the findings of low-to-normal TIBC, no low serum iron, and no between-group differences in TIBC, TSAT, or haemoglobin argue against absolute ID and instead support functional ID/inflammation-driven ferritin elevation. When ferritin was excluded from the ID definition, group differences disappeared, whereas TSAT <20% remained associated with long-term MACE.^11^ Thus, ferritin-based ID definitions in STEMI are prone to confounders, as inflammation-related ferritin elevation with iron status could potentially mislead ID in this context.

### Limitations

One of the most important limitations of this study is its retrospective, observational design introduces potential selection bias that cannot be completely ruled out despite the lack of basal statistical differences between groups. Second, ∼40% of patients were excluded due to missing ferritin values, limiting a bigger sample size and limiting adequate subgroup analyses. Third, as a single-centre study in a Latin American cohort, external validity is limited, necessitating validation in multicentre cohorts from diverse geographic and demographic populations. Fourth, patients with NSTEMI were not included; therefore, these findings should not be extrapolated to non-STEMI populations despite their known inflammatory component.

Additional methodological limitations that are worth further discussion are (1) absence of ferroptosis markers, soluble transferrin receptor levels and/or bone marrow biopsy to further assess ID; (2) lack of baseline troponin measurements and serial imaging studies preventing assessment of the relationship between ferritin and infarct size or LVEF; (3) use of the earliest available ferritin value (IQR time-to-measurement: 0–2 days) without serial measurements, which limits interpretation as higher values may reflect progressive inflammatory elevation rather than basal or peak levels; and (4) the observational design precludes determination of causality between ferritin and MACE risk, requiring mechanistic validation in future studies.

### Strengths

This study represents the largest study assessing the prognostic value of serum ferritin after a STEMI. Several methodological characteristics enhance the relevance of our findings. First, despite its retrospective design, no significant baseline differences were observed between groups stratified by pre-established ferritin cut-offs, controlling for potential confounders (*Table 1*). Second, the primary outcome remained independently significant after adjustment for established clinical prognostic variables and relevant inflammatory biomarkers, demonstrating ferritin's independent prognostic value. Third, the risk association was consistent across multiple time points, establishing both short- and long-term prognostic value (see Supplementary material online, *Figure S2*). Finally, restricted cubic spline analysis corroborated the pre-specified 300 ng/mL cut-off for the primary composite outcome, providing internal validation of the threshold effect (*Figure 5*).

### Future directions

These findings establish several important research priorities. First, future studies should comprehensively investigate iron metabolism pathways in cardiovascular disease beyond the standard ID definition in STEMI populations. Second, prospective validation studies should evaluate whether incorporating ferritin into existing clinical risk scores improves risk stratification for short- and long-term cardiovascular outcomes, particularly HF. Nonetheless, translational research is needed to define the specific pathophysiological mechanisms linking elevated ferritin to adverse outcomes. Key questions not possible to address by this study include whether elevated ferritin primarily reflects inflammatory burden vs. iron dysmetabolism and which specific pathway predominantly drives the observed outcomes. Understanding these specific mechanisms will be critical for identifying potential therapeutic targets and encourage the design of clinical trials evaluating specific therapeutic strategies. Potential interventions could include ferroptosis inhibitors, iron chelation therapy, strategies targeting myocardial iron metabolism, and anti-inflammatory approaches specifically modulating the ferritin pathway.

## Conclusions

Elevated baseline ferritin (≥300 ng/mL) independently predicts adverse cardiovascular outcomes in STEMI patients, providing incremental prognostic value for risk stratification beyond conventional markers. As the first STEMI-specific analysis of ferritin as a prognostic biomarker, this study establishes a foundation for future research to elucidate mechanisms. This study provides a basis for subsequent research aimed at clarifying underlying mechanisms, validating findings in diverse populations, and determining whether ferritin represents a modifiable therapeutic target to enable more precise, personalized approaches to post-MI care.

## Supplementary Material

oeag092_Supplementary_Data

## Data Availability

The data underlying this article will be shared on reasonable request to the corresponding author.
