# Supplementary material for: Admission ferritin in STEMI predicts long-term major adverse cardiovascular outcomes
Source: Eur Heart J Open. 2026 Jun 9;6(4):oeag092. doi: 10.1093/ehjopen/oeag092 (PMC13469897; doi:10.1093/ehjopen/oeag092)
Supplement: Supplementary file 1 [file oeag092_supplementary_data.docx]

**Supplementary material**

| **Ferritin ≥300 (ng/mL)** | **HR (CI 95%)** | ***p*** | **aHR (CI 95%)** | ***p*** |
| --- | --- | --- | --- | --- |
| **Follow-up 4.5 years** |  |  |  |  |
| MACE | 1.43 (1.06 to 1.94) | 0.01 | 1.47 (1.03 to 2.08) | 0.02 |
| Death | 1.99 (1.08 to 3.61) | 0.02 | - |  |
| New CHF | 1.47 (1.06 to 2.04) | 0.02 | - |  |
| Reinfarction | 0.90 (0.38 to 2.14) | 0.82 | - |  |
| Cardiogenic shock | 1.60 (0.90 to 2.84) | 0.10 | - |  |
| Mayor bleeding | 1.60 (0.78 to 3.30) | 0.19 | - |  |
| **Follow-up 1 year** |  |  |  |  |
| MACE | 1.21 (0.85 to 1.71) | 0.27 | 1.35 (0.91 to 2.01) | 0.13 |

**Table S1. Correlation of Ferritin Value with Primary Composite MACE Outcome and Individual Outcomes**

Presents unadjusted and adjusted ferritin risk at 4.5 and 1 year. Covariables for adjustment: albumin, NTpro-BNP, hsCRP, WBC, and GRACE score.

CHF = conhestive heart failure; MACE = major adverse cardiovascular events; aHR = adjusted HR. Statistical test: Cox proportional hazard; WBC = white blood cells

| **Covariable** | **b** | **SEM** | **Wald** | ***P*** | **Exp(b)** | **CI 95% of Exp(b)** |
| --- | --- | --- | --- | --- | --- | --- |
| **Ferritin ≥300 ng/mL** | 0.387 | 0.177 | 4.751 | 0.0293 | 1.4730 | 1.0398 to 2.0865 |
| **Albumin** | 0.221 | 0.181 | 1.489 | 0.2223 | 1.2482 | 0.8743 to 1.7821 |
| **NTpro-BNP** | -0.00002 | 0.00002 | 1.371 | 0.2415 | 1.0000 | 0.9999 to 1.0000 |
| **hsCRP** | -0.00003 | 0.0005 | 0.005 | 0.9411 | 1.0000 | 0.9989 to 1.0010 |
| **GRACE** | 0.003 | 0.002 | 2.391 | 0.1220 | 1.0040 | 0.9989 to 1.0090 |
| **Leukocytes** | -0.014 | 0.021 | 0.458 | 0.4984 | 0.9858 | 0.9458 to 1.0275 |

**Table S2. Hazard ratio adjustment by covariates**

Adjusted for albumin, NTpro-BNP, hsCRP, leukocytes, and GRACE.

hsCRP = high-sensitivity C-reactive protein; Statistical test: Cox proportional hazard

| **Variable** | **Ferritin <100 ng/mL**  (n=92) | **Ferritin 100-299 ng/mL**  (n=241) | **Ferritin ≥300 ng/mL**  (n=142) | ***p*** |
| --- | --- | --- | --- | --- |
| Age, years | 61.3 ± 1.08 | 58.1 ± 0.67 | 57.2 ± 0.93 | ns |
| Male, n (%) | 72 (78.2) | 217 (90.0) | 128 (90.1) | .007 |
| **Comorbidities and past medical history, n (%)** | | | | |
| T2D | 32 (35.16) | 81 (33.61) | 45 (31.91) | 0.87 |
| Hypertension | 41 (44.57) | 107 (44.40) | 68 (48.23) | 0.75 |
| Dyslipidemia | 40 (16.60) | 25 (17.73) | 25 (17.73) | 0.04 |
| CKD | 3 (3.26) | 3 (1.24) | 4 (2.84) | 0.40 |
| Obesity | 44 (18.26) | 36 (25.53) | 36 (25.53) | 0.08 |
| Smoking | 34 (36.96) | 112 (46.47) | 63 (44.68) | 0.29 |
| Previous MI | 11 (11.96) | 18 (7.47) | 18 (12.77) | 0.18 |
| Previous HF | 1 (1.09) | 3 (1.24) | 0 (0.00) | 0.42 |
| Atrial fibrillation | 1 (1.09) | 1 (0.41) | 0 (0.00) | 0.45 |
| **Clinical presentation** | | | | |
| GRACE score | 119.6 ± 4.0 | 142.4 ± 17.2 | 128.3 ± 2.99 | ns |
| Hemoglobin (IQR), g/dL | 15.7 ± 0.19 | 15.4 ± 0.12 | 15.4 ± 0.16 | ns |
| WBCs (IQR), 10^3^/uL | 11.9 ± 0.42 | 12.2 ± 0.31 | 12.0 ± 0.36 | ns |
| Glucose (IQR), mg/dL | 178 ± 8.96 | 203 ± 7.20 | 189 ± 7.86 | ns |
| Creatinine (IQR), mg/dL | 1.166 ± 0.10 | 1.12 ± 0.06 | 1.10 ± 0.04 | ns |
| NTpro-BNP (IQR), pg/mL | 1652 ± 388.7 | 2698 ± 290.1* | 2620 ± 416.3* |  |
| hsCRP (IQR), mg/L | 18.32 ± 4.07 | 36.32 ± 5.27 | 32.51 ± 5.13 | ns |
| Albumin (IQR), g/dL | 3.71 ± 0.05 | 3.61 ± 0.03 | 3.69 ± 0.03 | ns |
| Ferritin (IQR), ng/mL | 58.9 ± 2.77*** | 192.7 ± 3.72*** | 896.8 ± 150.7 |  |
| Ferritin (IQR), days since admission | 0.83 ± 0.09 | 1.1 ± 0.06 | 1.4 ± 0.11 | ns |

**Table S3. Baseline Characteristics of the Population. Ferritin Groups <100, 100-299, and ≥300 ng/mL**

*P<0.0001 vs <100 ng/mL

**P<0.0001 vs 100-299 ng/mL

***P<0.0001 vs >300 ng/mL

n = number; IQR = interquartile range; HbA1C = glycated hemoglobin; hsCRP = high-sensitivity C-reactive protein; CKD = chronic kidney disease; T2D, type 2 diabetes; WBCs, white blood cells. Statistical tests: Chi-square and Mann-Whitney Statistical tests: Chi-square and ANOVA

**Figure S1. Distribution of follow-up duration.** **(Left)** Frequency histogram showing the temporal distribution of patient follow-up. The superimposed dashed curve represents the theoretical normal distribution (p<0.0001 to reject Normality). **(Right)** Complementary Cumulative Distribution Function plot illustrating the probability of patients remaining in follow-up over time (from 100% to 0%). The dashed line represents the expected curve for a normal distribution based on the cohort’s mean and standard deviation.

Statistical analysis for normal distribution: D`Angostino-Pearson test.

**Figure S2. Kaplan-Meier Curve of MACE Events with Analysis at 30 Days, 90 Days, and 2.5 Years. Ferritin <300 (blue) and ≥300 ng/mL (red)**

d = days; MACE = major adverse cardiovascular events; n = number. Statistical analysis: Cox proportional hazard

**Figure S3. Landmark analysis of MACE at 30 Days and >30 Days for Ferritin. Kaplan-Meier Curve of MACE Events at 30 Days and >30 Days for Ferritin Groups <300 (blue) and ≥300 ng/mL (red)**

MACE = major adverse cardiovascular events, n = number; CI = confidence interval. Statistical test: Cox proportional hazard

**Figure S4. Kaplan-Meier Curve of MACE Events at 1 year. Ferritin <300 (blue) and ≥300 ng/mL (red)**

MACE = major adverse cardiovascular events; n = number. Statistical analysis: Cox proportional hazard

**Figure S5. MACE at 1 Year for Iron Deficiency. Kaplan-Meier Curve of MACE Events at 1 Year Follow-up for Groups Without Iron Deficiency (blue) and With Iron Deficiency (red)**

MACE = major adverse cardiovascular events, n = number. Statistical test: Cox proportional hazard
